# Orthogonal binding and displacement of different guest types using a coordination cage host with cavity-based and surface-based binding sites†

**DOI:** 10.1039/d1sc04272f

**Published:** 2021-08-25

**Authors:** Michael D. Ludden, Christopher G. P. Taylor, Michael D. Ward

**Affiliations:** Department of Chemistry, University of Warwick Coventry CV4 7AL UK m.d.ward@warwick.ac.uk

## Abstract

The octanuclear Co(ii) cubic coordination cage system **H** (or **HW** if it bears external water-solubilising substituents) has two types of binding site for guests. These are (i) the partially-enclosed central cavity where neutral hydrophobic organic species can bind, and (ii) the six 'portals' in the centres of each of the faces of the cubic cage where anions bind *via* formation of a network of CH⋯X hydrogen bonds between the anion and CH units on the positively-charged cage surface, as demonstrated by a set of crystal structures. The near-orthogonality of these guest binding modes provides the basis for an unusual dual-probe fluorescence displacement assay in which either a cavity-bound fluorophore (4-methyl-7-amino-coumarin, **MAC**; *λ*_em_ = 440 nm), or a surface-bound anionic fluorophore (fluorescein, **FLU**; *λ*_em_ = 515 nm), is displaced and has its emission ‘switched on’ according to whether the analyte under investigation is cavity-binding, surface binding, or a combination of both. A completely orthogonal system is demonstrated based using a **Hw**/**MAC**/**FLU** combination: addition of the anionic analyte ascorbate displaced solely **FLU** from the cage surface, increasing the 515 nm (green) emission component, whereas addition of a neutral hydrophobic guest such as cyclooctanone displaced solely **MAC** from the cage central cavity, increasing the 440 nm (blue) emission component. Addition of chloride results in some release of both components, and an intermediate colour change, as chloride is a rare example of a guest that shows both surface-binding and cavity-binding behaviour. Thus we have a colourimetric response based on differing contributions from blue and green emission components in which the specific colour change signals the binding mode of the analyte. Addition of a fixed red emission component from the complex [Ru(bipy)_3_]^2+^ (**Ru**) provides a baseline colour shift of the overall colour of the luminescence closer to neutral, meaning that different types of guest binding result in different colour changes which are easily distinguishable by eye.

## Abstract 2

Orthogonal binding of neutral or anionic fluorophores to the cavity or surface, respectively, of a coordination cage host allows a dual-probe displacement assay which gives a different fluorescence colorimetric response according to where analyte species bind.

## Graphical abstract

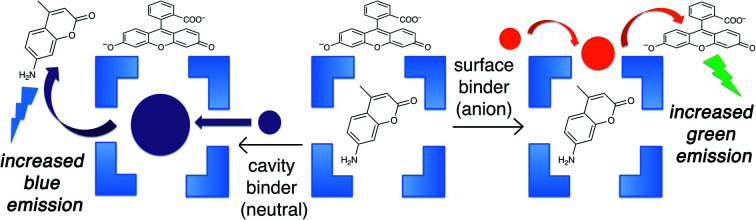

## 1 Introduction

Coordination cages – hollow, pseudo-spherical metal/ligand assemblies – are well known to be able to bind small molecular guests in their central cavities,^1^ with a range of consequences for, and potential applications in, areas such as catalysis,^2^ sensing^3^ and transport.^4^ The focus on guest binding has mostly been occupancy of these central cavities, as they present an obvious binding site with well-defined size, shape and (sometimes) functional group characteristics which obviously relate to their molecular recognition properties.

**Fig. 1:**
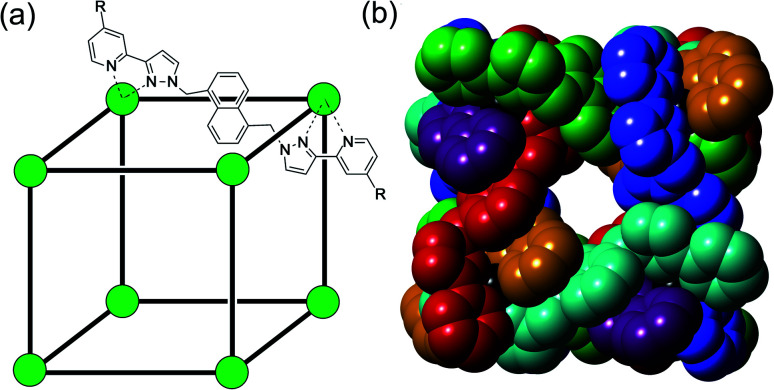
The host cage [Co_8_L_12_]^16+^, abbreviated as **HW** (R = CH_2_OH) or **H** (R = H). (a) A sketch emphasising the cubic array of Co(ii) ions and the disposition of one bridging ligand; (b) a space-filling view of the core (without the CH_2_OH substituents) showing each ligand coloured separately for clarity.

More recently, we^5,6^ and others^7,8^ have noticed that the exterior surfaces of cages also provide recognition sites for, principally, ionic guests. The same characteristics of the interior surface of a cage (hydrophobicity arising from organic ligand components; charge density arising from metal ion vertices; possibly the presence of functional groups) that facilitate guest binding can also exist at the exterior surfaces. Although – by definition – the exterior surfaces are not enclosed and do not provide the clearly defined three-dimensional cavities that the interior surfaces provide, they still offer opportunities for cages to interact with small molecules or ions. In particular, in our octanuclear M_8_L_12_ cubic cage family **H**/**HW** (Fig. 1), the portals in the cage faces provide a preorganised cyclic array of multiple weak CH hydrogen-bond donors from the ligand array, and – according to crystallographic evidence obtained with many different anions – these converge on an anion that is located in the portal.^5*a*^

Thus the **H**/**HW** cages combine both a central cavity for binding of hydrophobic organic guests, whose binding strength correlates with hydrophobic surface area;^9^ and surface-binding sites for anions, with the binding strength of the anion correlating with the ease of desolvation of the anion.^6^ A recent example of the importance of this combination is the efficient cage-based catalysis of the Kemp elimination reaction of benzisoxazole with hydroxide to give 2-cyanophenolate, using the cage **HW** as the catalyst.^10^ The cage-based catalysis (>10^5^ fold rate enhancement, depending on pH) relies on a shell of surface-bound and hence partially desolvated hydroxide ions, attracted to the portals around the cage surface by the high positive charge of the cage, being brought into close proximity to the neutral benzisoxazole guest which is bound in the central cavity. Thus, cavity-binding of a neutral hydrophobic guest, and electrostatically-driven surface-binding of hydroxide ions, cooperate very effectively to promote catalysis by co-locating the two reaction partners using different interactions: even under weakly basic conditions (pH 8 in bulk solution) the accumulation of hydroxide ions at the cage surface, surrounding the cavity-bound guest, is such that the *local* pH in the cavity is effectively 13–14.^5*a*^

We have used fluorescence-based methods to investigate both types of guest binding associated with **HW**, as the cage quenches fluorescent guests which bind either in the cavity or at the surface-based sites. Thus 4-methyl-7-amino-coumarin (**MAC**) binds in the central cavity in water, principally due to its hydrophobicity, and its fluorescence is quenched by the nearby Co(ii) ions.^9*a*^ Similarly the di-anionic fluorophore fluorescein (**FLU**) is an exterior surface binder, anchored to the portals in the six face centres, and is likewise quenched on binding by proximity to the Co(ii) ions.^6^ These effects have been used independently as the basis of two different types of fluorescence displacement assay (FDA) – a measurement of a binding constant in which the analyte under evaluation competitively displaces a fluorescent (but quenched) indicator, whose binding constant is known. The binding of the analyte can then be evaluated from how much of the fluorescence from the indicator is restored when it is displaced by the analyte which competes for the same binding site.^11^ In the first case binding of neutral organic guests in the cavity of **HW** displaces (and restores fluorescence from) a cavity-bound **MAC** molecule, which allows binding of a wide range of non-chromophoric guests to be evaluated.^9*a*^ In the second case displacement of **FLU** from the cage surface by a range of simple organic and inorganic anions restored its florescence, likewise allowing binding constants of the various anions for the surface binding sites to be evaluated.^6^ Such analyses have allowed us to probe independently the affinities of different guests for either cavity- or surface-based binding sites on the same cage host using quite distinct interactions.

Accordingly in this paper we report further studies, both crystallographic and solution-based, into the ability of the **H**/**HW** cage system to participate in cavity-based and surface-based binding of different types of guest independently of one another. In the first part of the paper we report crystallographic studies showing how ‘crystalline sponge’ experiments can be performed with a combination of cavity-based and surface-based guests, to introduce a guest into the cavity of a host cage **H** and also to change the anion shell surrounding it, in single-crystal to single-crystal transformations.^12^ In the second part of the paper we describe how the two different types of FDA can be combined in a single analytical process, allowing evaluation of the ability of specific guests to occupy the cavity or surface sites of **HW** in solution a single experiment, giving a unique colorimetric response according to how much of the distinct cavity-bound and surface-bound fluorescent indicators are displaced by a particular type of analyte. Whilst there have been some examples of displacement assays that use different spectroscopic measurements on the same displaced dye to report on different aspects of guest binding (*e.g.* both concentration and enantiomeric excess of a chiral amine binding),^13^ we believe this to be the first example of such an assay based on independent displacements of two different fluorescent indicators that are bound in different ways to the same host.

These demonstrations of the independence of the two types of cage/guest interaction, both in crystalline sponge experiments and in solution, will in addition extend our ability to develop new supramolecular catalysts. Given that the potential generality of this type of cage-based catalysis is driven by the ability to surround any cavity-binding organic guest with a high local concentration of any surface-binding ion,^5*a*^ being able to control binding of both components independently of one another will be the basis of further progress in identifying catalytic process that can be mediated by the cage.

## 2 Results and discussion

### 2.1. Crystalline sponge experiments showing combined guest uptake and anion exchange

We have reported many examples of cage/guest complexes that were prepared using the crystalline sponge method, whereby X-ray quality single crystals of the cage **H** were soaked in a pure organic guest (if the guest is an oil), or a concentrated solution of the guest in MeOH, for several hours. This can result in uptake of guest into the cage cavity without loss of crystallinity.^5*a*,14^ As-prepared crystals of **H** contain a network of methanol molecules in the cage cavity, and BF_4_^−^ counter-anions, some of which occupy the portals around the cage surface.^15^ Interestingly, although the counter-ions occupy the portals and apparently block access to the cage interior, guests are still taken up into the cavity during these crystalline sponge experiments which implies that a considerable degree of dynamic behaviour in the crystals is possible at room temperature without loss of crystallinity. Crystals of **H** can also undergo anion-exchange in the same way, with immersion of **H** crystals in concentrated methanolic Bu_4_NI resulting in the fluoroborate anions surrounding the cage being replaced by iodide anions in the cage portals.^5*b*^ Thus we have demonstrated how crystalline sponge experiments with **H** can be used to introduce either cavity-bound guests or to replace surface-bound anions. Here, we show that both can be accomplished in a single experiment.

Single crystals of **H** (tetrafluoroborate salt) prepared by the usual solvothermal method^15^ were soaked for several hours in concentrated methanolic solutions containing both a known cavity-binding guest (**MAC**) and the tetrabutylammonium salt of the desired replacement anion (iodide, nitrate, hexafluorophosphate, triflate, sulfate). Subsequent X-ray diffraction experiments confirmed that in some cases *both* types of guest had been taken up, with **MAC** occupying the cage cavity and the new anion type displacing tetrafluoroborate from the binding sites around the cage surface.

**Fig. 2:**
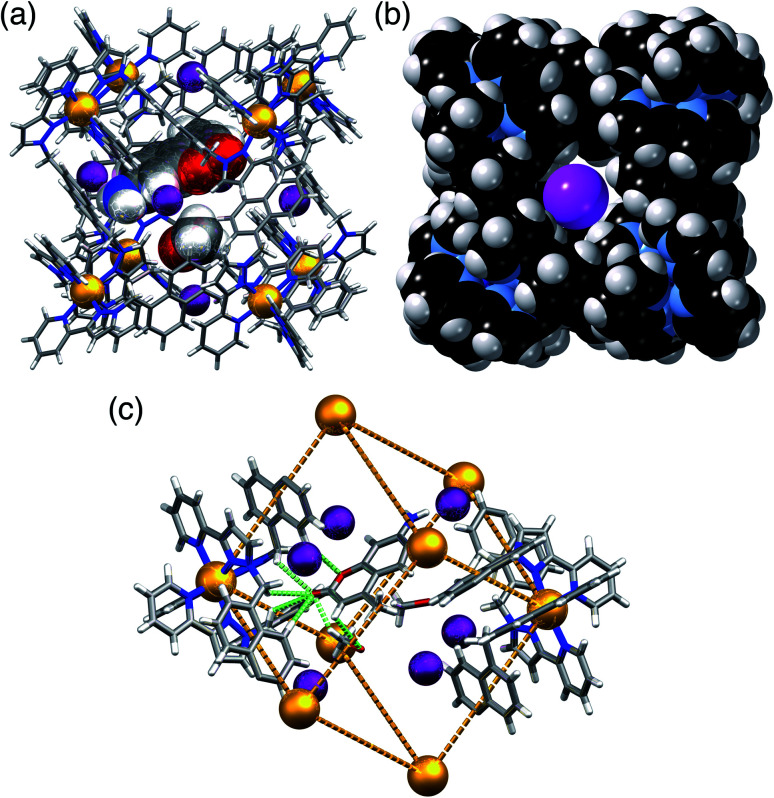
Views of the crystal structure of **H·MAC·I**. (a) A view of the complete cage showing the array of six surface-bound iodide anions (purple) as well as the cavity-bound guests (one MAC and one MeOH) shown space-filling. (b) A space-filling view of the cage looking onto one of the faces, emphasising the binding of the iodide anion I(2) (which has 100% occupancy) in the surface portals. (c) Partial view of the cage host, showing the six iodide guests, and showing the H-bonding interactions between MAC and the cage interior surface (green dashed lines indicate CH⋯O contacts with H⋯O separations of <3 Å).

In **H·MAC·I** (Fig. 2) the cage cavity contains one **MAC** guest and a pair of MeOH molecules (25% fractional site occupancy each in the asymmetric unit which consists of one half of the cage: hence one MeOH total), mutually disordered over the inversion centre at the centre of the cage cavity; additionally, iodide anions have replaced the fluoroborate anions in the portals around the cage surface (Fig. 2a). As usual the cavity-binding neutral guest molecule displays H-bonding interactions with the H-bond donor pockets of the cage interior: these pockets are formed at the two *fac* tris-chelate vertices at opposite ends of the cavity, by a convergent collection of CH protons which lie close to a Co(ii) ion and are therefore in a region of high positive electrostatic potential.^16^ Given the disorder of the **MAC** guest with the MeOH molecules a detailed analysis of the H-bonding interactions is inappropriate but the presence of multiple CH⋯O interactions, with H⋯O distances in the range 2.5–2.8 Å between cage interior surface and electron rich regions of the **MAC** guest, is clear and these are emphasised in Fig. 2c. Some of the iodide ions around the cage surface likewise display disorder over two closely-spaced positions, but atom I(2) has 100% occupancy, and the space-filling view in Fig. 2b shows how nicely this iodide ion sits in the portal on the 16+ cage surface, surrounded by CH protons from the ligands. Iodide is of course a weak hydrogen-bond acceptor.^17^ Many of the H⋯I distances in this cyclic array are longer than the sum of the van der Walls radii and therefore constitute very weak interactions, but the I(2)⋯H(13A) distance is 3.03 Å and some others are in the range 3.1–3.2 Å. Overall we could explicitly locate 13.6 iodide anions per 16+ complex cation. It is worth pointing out that with tetrafluoroborate rather than iodide as the counter-anion, the crystalline sponge experiment to absorb **MAC** results in uptake of a stacked pair of guests into the cage cavity.^14^ In this new structure of **H·MAC·I** however, one of the surface-bound iodide ions forms an OH⋯I hydrogen-bonding interaction with one of the MeOH molecules in the cavity (O⋯I separation 3.34 Å); hence the nature of the surface bound ion is having an effect on what is bound into the central cavity in the crystalline state.

**Fig. 3:**
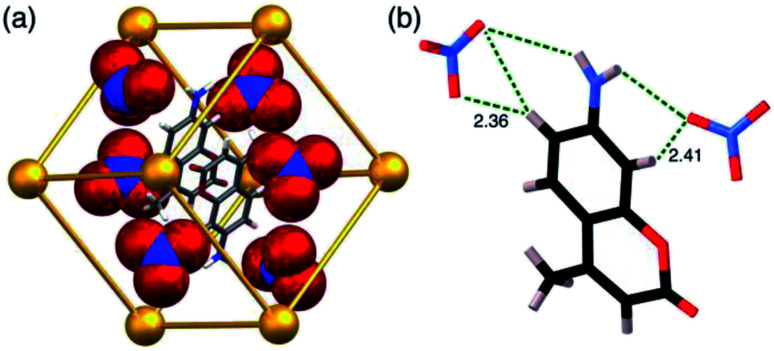
Views of the crystal structure of **H·MAC·(NO3)**. (a) A view showing the metal ions that define the cubic cage, the **MAC** guests, and the array of six nitrate anions around the surface (shown space-filling); (b) a view showing CH⋯O and NH⋯O interactions between a **MAC** guest and two of the surrounding nitrate anions.

**H·MAC·(NO3)** (Fig. 3) behaves similarly to the iodide salt, in that there is one **MAC** guest with 0.55 site occupancy, disordered with a MeOH molecule having a site occupancy of 0.35 on the other side of the cavity. This means a total average occupancy of 1.1 **MAC** guests and 0.7 MeOH guests, requiring that some cage molecules in this crystalline sample contain a stacked pair of **MAC** guests: *e.g.* we could have 55% of the molecules in the crystal containing a stacked pair of **MAC** guests and the remaining 45% of the molecules containing only MeOH. The anion exchange from BF_4_^−^ to nitrate is incomplete, with the lattice containing 13 nitrate ions and three BF_4_^−^ anions per cage complex cation; however the positions in the cage portals are all occupied by the nitrate anions (Fig. 3a). Again we see hydrogen-bonding interactions between cavity-bound and surface bound guests: Fig. 3b highlights the CH⋯O and NH⋯O interactions between the **MAC** guest and the surface-bound nitrate anions. The nitrate anions also form a network of CH⋯O contacts (≥2.5 Å for the O⋯H distances) with CH units from the ligand array around the portals in the same way as seen for the other anions.

**Fig. 4:**
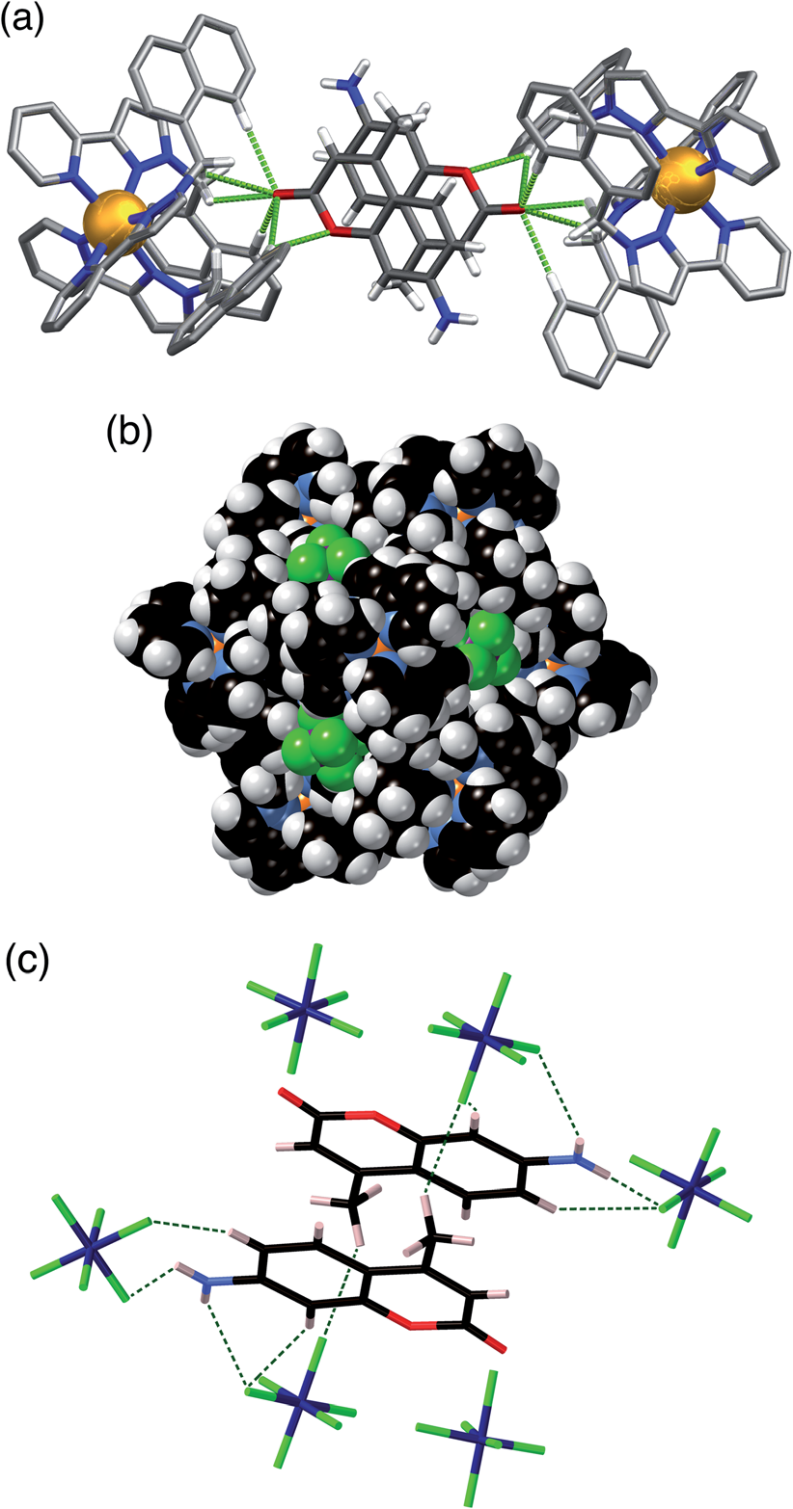
Views of the crystal structure of **H·MAC·(PF6)**. (a) A view showing the stacked pair of **MAC** guests with part of the surrounding cage, emphasising the H-bonding interactions between inwardly-directed CH protons on the cage surface and the **MAC** guests: CH⋯O interactions with H⋯O distances of <3 Å are shown by green dotted lines. (b) A space-filling view of the cage looking onto one of the metal vertices, emphasising the binding of the hexafluorophosphate anions (green) in the surface portals. (c) The array of six hexafluorophosphate anions surrounding the two cavity-bound **MAC** molecules, with short (<3 Å) H⋯F contacts between **MAC** and [PF_6_]^−^ anions arising from CH⋯F or NH⋯F interactions shown by green dotted lines.

A similar experiment using **MAC** as the cavity-bound component coupled with Bu_4_NPF_6_ to provide anion-exchange affords **H·MAC·(PF6)**. Again we see a set of six surface-bound anions surround in the central cavity, but the cavity now contains a stacked pair of **MAC** guests (Fig. 4) which have site occupancies of 1 each, *i.e.* two per cage cavity, so the occupancy in the crystal is complete. The arrangement of this stacked guest pair is very similar to what we have reported before,^14^ although in this case the two guests are crystallographically inequivalent rather than being related by inversion. The H-bonding of each **MAC** carbonyl group with the H-bond donor pockets on the cage interior is clear with multiple CH⋯O contacts having H⋯O separations as low as 2.44 Å, with all such contacts of <3 Å highlighted in Fig. 4a. The hexafluorophosphate anions again occupy the cage portals, making multiple CH⋯F contacts with the surrounding ligand array of which the shortest is 2.43 Å (Fig. 4b). The view in Fig. 4c emphasises how the octahedral array of anions surrounds and interacts with the stacked pair of cavity-bound guests. Specifically each **MAC** guest has outwardly-directed CH (two aromatic, plus one from the methyl group) and NH protons which project towards the portals and form H⋯F contacts with F atoms of nearby hexafluorophosphate anions, with H⋯F contacts as short as 2.33 (for an NH⋯F contact) and 2.48 Å (for a CH⋯F contact). Given the positional disorder of some of the F atoms in the hexafluorophosphate anions these distances should not be over-analysed: but the general picture of multiple H-bonding contacts between cavity-bound guests and the surface-bound anions, as shown in Fig. 4c, is clear: and we note that this accessibility of cavity-bound guests to surface-bound anions underpins the catalytic activity that we observed in previous studies.^5,10^

**Fig. 5:**
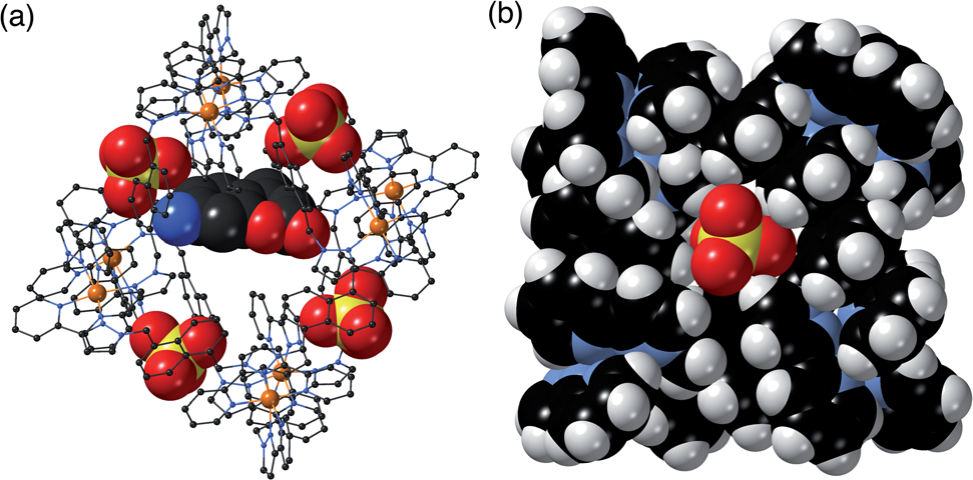
Views of the crystal structure of **H·MAC·(SO4)**. (a) A view of the complete cage in wireframe showing the four surface-bound sulfates in space-filling mode, with a space-filling **MAC** guest included; (b) a space-filling view onto one face showing how the sulfate anion occupies the portal in the centre of the face *via* multiple CH⋯O contacts.

**H·MAC·(SO4)** (Fig. 5) has guest occupancies similar to those of the iodide salt, with site occupancies of 0.5 per **MAC** and 0.5 per MeOH in each half of the cage, summing to one of each per cage. This is the first instance of a structurally-characterised cage of this family containing a dianion. The sulfates behave similarly to the mono-anions described above in that some of them occupy portals on the cage surface surrounding the cavity-bound guest, participating in CH⋯O contacts with the ligands that define the portals (Fig. 5). However only four of the six portals contain an embedded sulfate anion (Fig. 5a), which are involved in multiple CH⋯O interactions with H⋯O distances down to 2.5 Å with the surrounding ligand array. In addition, there are some hydrogen-bonding contacts between a surface-bound sulfate and cavity-bound **MAC** guest with a CH⋯O interaction involving an aromatic CH of the guest having an H⋯O separation of just 2.33 Å, and an NH⋯O interaction with the coumarin amine group having an H⋯O separation of 2.67 Å. The **MAC** guest shows the usual H-bonding interactions with the cage interior surface that have been described before.

**Fig. 6:**
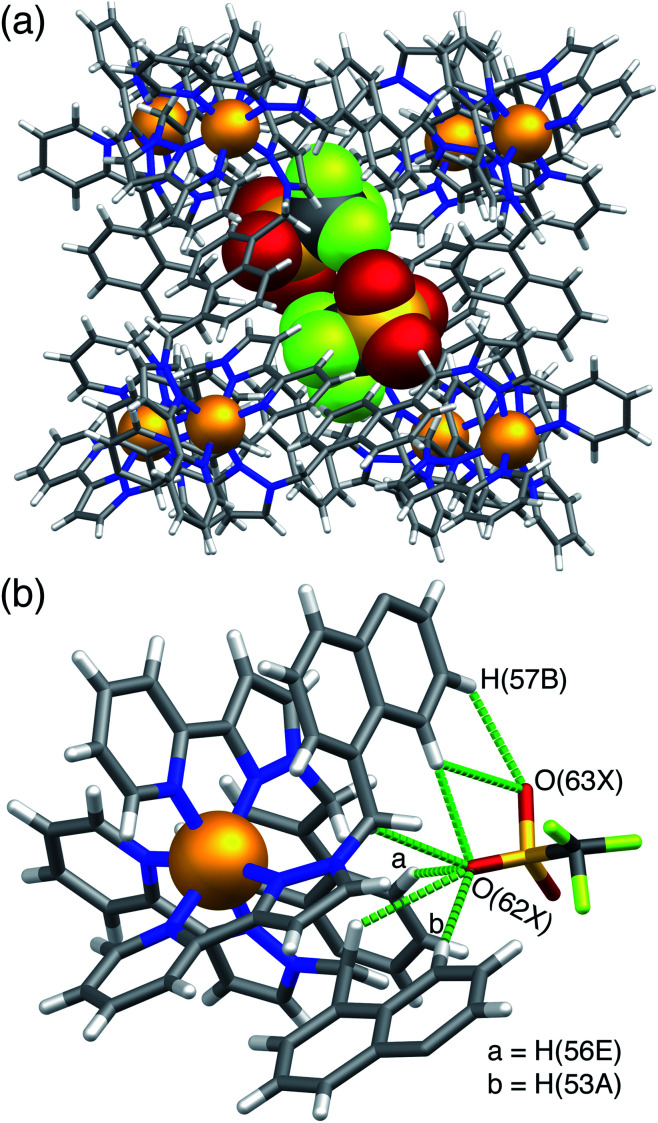
Views of the crystal structure of **H·(CF3SO3)** (F, green; O, red; S, yellow). (a) A view of the complete cage in wireframe showing the two half-occupancy triflate anions in space filling mode; (b) a view showing the hydrogen-bonding interactions of the triflate anion with the interior surface of the cage around one of the *fac* tris-chelate vertices, with H⋯O contacts of <3 Å shown by purple dashed lines.

**H·(CF3SO3)** is unusual, and particularly significant for the purposes of this work, in that it contains both cavity-bound as well as exterior anions; the presence of the cavity-bound triflate anion precludes binding of **MAC** which is not present in this structure although it was available in the crystal soaking experiment. Fig. 6a shows a view of the cage containing a triflate anion, disordered over two equivalent positions astride the inversion centre with 0.5 site occupancy in each position. Two of the O atoms [O(62X) and O(63X)] project into the H-bond donor pocket at the *fac* tris-chelate site around Co(2) that is defined by the convergent set of naphthyl CH and methylene CH_2_ protons, such that there are – in the usual way – multiple CH⋯O hydrogen bonds between encapsulated anion as H-bond acceptor and the cage surface which acts as the H-bond donor, with H⋯O contacts down to 2.50 Å [O(62X)⋯H(53A)], 2.61 Å [O(62X)⋯H(56E)] and 2.72 Å [O(63X)⋯H(57B) (Fig. 6b). The 0.5 site occupancy of each triflate could mean that half of the cage cavities have taken up a pair of anions in the crystalline sponge process, but this would result in unfeasibly short inter-anion contacts (a 2.39 Å O⋯F contact which is significantly less than the sum of the van der Waals radii). This strongly implies that each cage contains one cavity-bound triflate anion that is randomly located in one of the two equivalent off-centre positions. The lattice contains an additional crystallographically distinct cage complex unit (lying on a threefold symmetry axis rather than astride an inversion centre) and this also contains a triflate ion but the disorder there is far more severe so the geometry cannot be discussed in detail. Triflate anions outside the cavity form a range of O⋯HC contacts with the cage exterior surface: but the important point to emphasise is that this is the first observation of a cavity-bound anion in any of the crystal structures we have obtained with this cage system.

The overall messages from this set of related structures are that (i) simultaneous exchange of both cavity-based (neutral) and surface-bound (anionic) guests is possible in a single crystalline sponge experiment, in addition to the possibilities, reported earlier,^5,9,10,14^ of introducing each guest type on its own; and (ii) we can see in several cases close contacts between the cavity-bound guests and the surrounding anion array in the form of short CH⋯X contacts. It follows from this that binding of the two guest types may not be genuinely orthogonal events, *i.e.* wholly independent of one another, although the results of the displacement assays reported in the next section show that this is approximately true in some cases and forms the basis of the two-indicator displacement assay.

### 2.2. Independent displacement of cavity-bound and surface-bound fluorophores by different guests

#### 2.2.1 Testing the two types of fluorescence displacement assay individually

**Chart 1:**
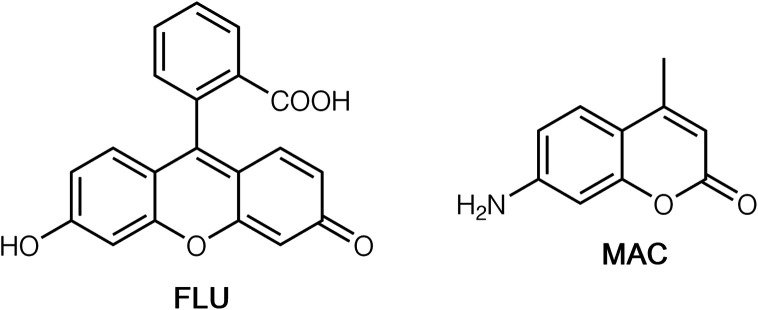
Structural formulae of **FLU** and **MAC**.

Given the ability of the cage to accommodate cavity-bound and surface-bound guests independently of one another, we wished to investigate the possibility of each guest type being independently addressable in a FDA, with each guest type being displaced independently according to the nature of the analyte. The obvious choice for the cavity-binding fluorophore, based on our previous work, is **MAC** (Chart 1; *λ*_em_ 445 nm, blue), given its strong binding inside **Hw** in water,^9*a*^ the structural characterisation of examples of cage/**MAC** complexes,^14^ and the fact that it has already been used in a FDA to evaluate binding strengths of other cavity-binding guests.^9*a*^ Likewise, the obvious choice for a fluorescent surface-bound anion is fluorescein, **FLU** (Chart 1; *λ*_em_ 515 nm, green) whose strong binding to the surface of **Hw** and its consequent use in a displacement assay for evaluating relative binding strengths of other anions has also recently been described.^6^ Importantly, the cavity-binding fluorophore (**MAC**)^9*a*^ and the surface-binding fluorophore (**FLU**)^6^ have different fluorescence colours such that displacement of different amounts of each according to where the guest binds to **HW** should provide a diagnostic colorimetric response. Note that although we used unsubstituted cage **H** for the crystalline sponge X-ray diffraction experiments reported above because of the ease of growth and robustness of its single crystals,^14^ for solution studies in water we used **Hw** whose twenty-four hydroxymethyl substituents render it water-soluble: apart from these exterior groups the two are isostructural with essentially identical cavities.^9*b*^

We started by using **MAC** or **FLU** as fluorescent reporters of guest binding, as we have done before, but added one variation to the process.^6,9*a*^ Although we can calculate binding constants from titrations which displace these guests and result in a growth in fluorescence intensity, a simple change in fluorescence intensity alone is of limited value as an analytical tool without careful calibration: and this is particularly true where a naked-eye test for substrate binding is sought as the human eye is poor at estimating absolute light intensity values. We could however convert this change in fluorescence intensity of one component to a ratiometric response by adding to the system a fixed amount of the red-luminescent species [Ru(bipy)_3_]Cl_2_ (denoted **Ru**). The dication of **Ru** will not associate with the 16+ cation of **HW** in solution: but the presence of a fixed red luminescence component as a baseline means that during the titrations with cavity-binding or surface-binding guests which liberate **MAC** or **FLU** respectively, the steady increase in the blue or green luminescence component combines with the fixed red luminescence component of **Ru** to give an obvious change in the overall hue – *i.e.* the intensity-only change in one emission component is converted to a ratiometric response as the balance between the fixed (red) and variable (blue or green) emission changes. This is a technique that has been used elsewhere to convert an intensity-based luminescence change into a ratiometric change for sensing applications.^18^

**Fig. 7:**
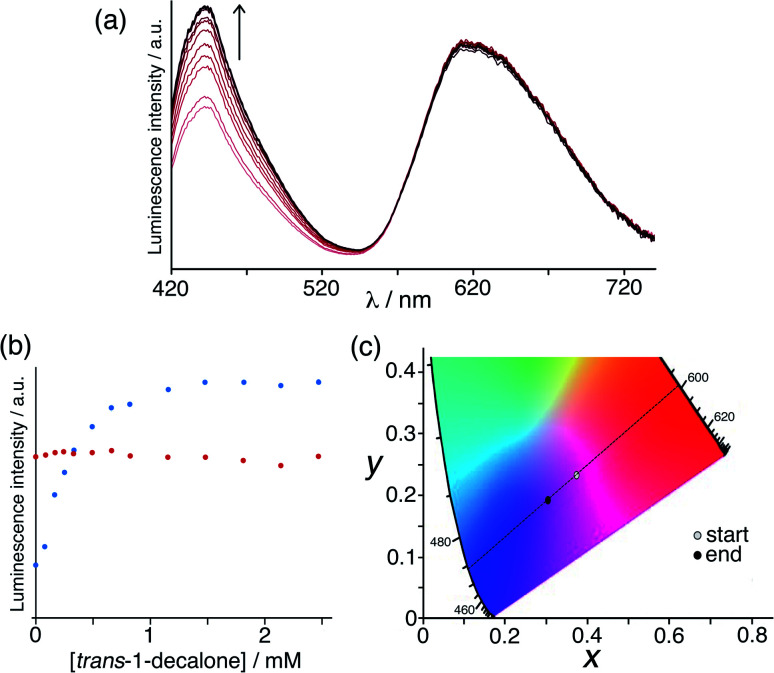
Results of titration of the cavity-binding guest *trans*-1-decalone into a combination of **HW** (150 μM), **MAC** (10 μM) and **Ru** (50 μM) in water. (a) Fluorescence changes associated with MAC being displaced from the cage cavity (*λ*_exc_ = 395 nm); (b) plot of the peak intensity data from part (a) during the titration; (c) shift in overall emission colour on a CIE colour-space chart during the titration.

Initially we used the host cage **HW** (150 μM), **MAC** (10 μM) and **Ru** (50 μM) as the sensor system. With these proportions the steady-state luminescence spectrum shows the characteristic blue emission maximum of residual free **MAC** (the fraction that is not quenched by binding to inside **HW**) and the red phosphorescence of **Ru** at *ca.* 620 nm. On addition of portions of the hydrophobic, cavity-binding guest *trans*-1-decalone, we observed (Fig. 7a) a progressive increase in the **MAC** fluorescence component as this is displaced, whereas the **Ru**-based emission component was essentially invariant. Fitting the rise in **MAC** fluorescence (Fig. 7b) to a 1 : 1 binding isotherm using the same software as reported previously,^9*a*^ that takes account of competition between **MAC** and the *trans*-1-decalone, we obtained a *K* value of guest binding of 3 × 10^4^ M^−1^: this is slightly higher than the value of 1 × 10^4^ M^−1^ reported earlier which may be ascribed to the different experimental conditions.^9*a*^ Significantly however there is now an overall colour change associated with adding increased blue fluorescence to a fixed red phosphorescence background, which is shown on a CIE colour space diagram in Fig. 7c as an overall colour shift towards purple.

**Fig. 8:**
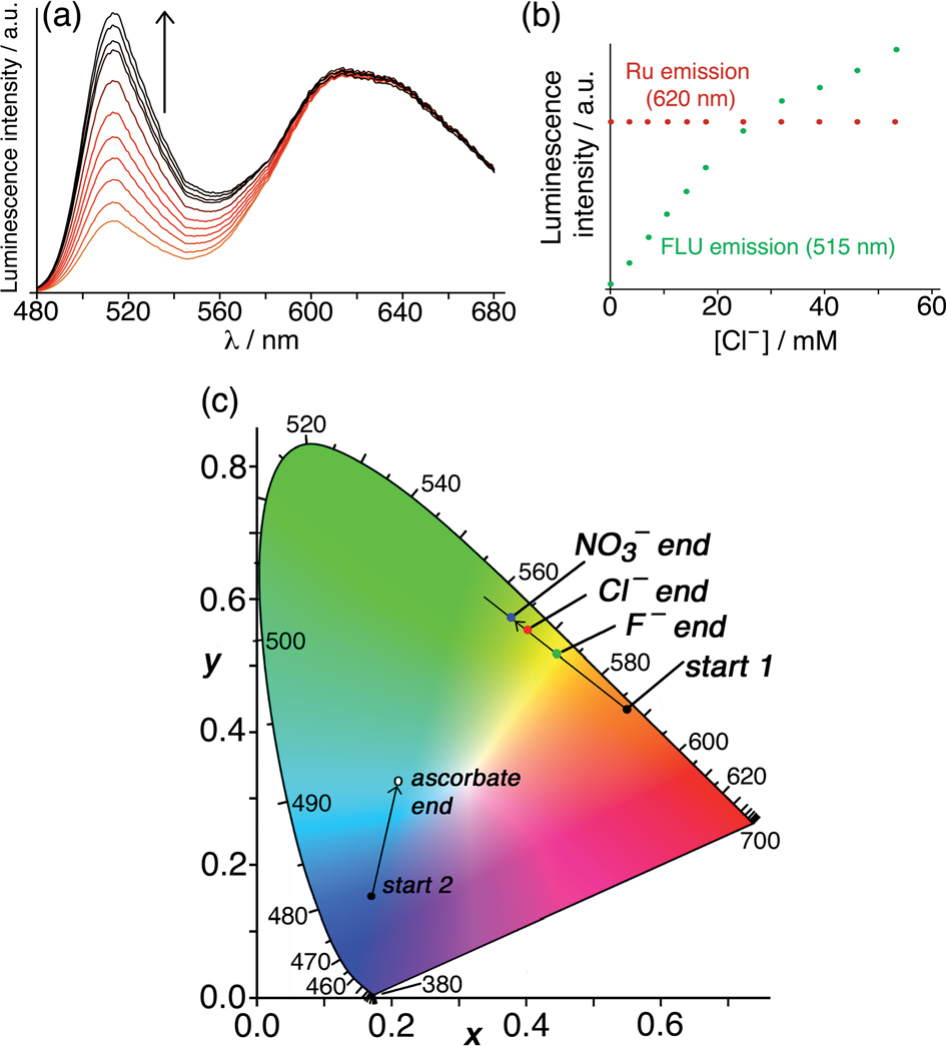
Results of titrations of the surface-binding anionic guests chloride, fluoride and nitrate into a combination of **HW** (130 μM), **FLU** (10 μM) and **Ru** (50 μM) in water. (a) Fluorescence changes associated with **FLU** being displaced from the cage surface during titration with chloride (*λ*_exc_ = 450 nm); (b) plot of the peak intensity data of both emission components from part (a) during the titration; (c) shift in overall emission colour (beginning from ‘*start 1*’) on a CIE colour-space chart during similar titrations with nitrate, chloride and fluoride, confirming that nitrate binds most strongly of these three anions to the cage surface due its hydrophobicity. (Note that the data relating to addition of ascorbate [beginning from ‘*start 2*’] relate to the titrations in Fig. 10, *q.v.*)

A similar experiment to probe surface anion binding, and convert it to a visible colorimetric response, was conducted using **HW** (130 μM), **FLU** (10 μM) and **Ru** (50 μM). The steady-state emission spectrum showed the expected broad, red **Ru**-based emission centred at 620 nm and a green emission component at 515 nm associated with the small proportion of un-quenched **FLU**. Titration of portions of NaCl, NaF or NaNO_3_ into this solution (Fig. 8a illustrates the effect of added chloride) resulted in displacement of **FLU** from the cage surface by the added ions and an increase in the green emission component only (Fig. 8b), with the most hydrophilic anion (fluoride) showing the smallest effect.^6^ When combined with the fixed red emission from **Ru**, the increased green emission from displaced **FLU** results in an overall colour change from orange to yellow as illustrated on the CIE diagram in Fig. 8c. As we might expect – but it is still nonetheless pleasing – the magnitude of the luminescence colour shift on the CIE diagram that is induced by the three different anions correlates with how well these anions bind to the cage surface (Fig. 8c),^6^ with nitrate having the largest effect and fluoride the smallest.

#### 2.2.2 Combining the two types of fluorescence displacement assay in a single system

Having confirmed the previously-established behaviour of the two sensing modalities using cavity-binding and surface-binding guests, but converting the outputs to a ratiometric change between two differently-coloured emission components, the next step was to see if we could combine these in one system. The goal is to have a single sensor system that responds to both cavity-binding and surface-binding analytes, giving a different colour response for each.

**Fig. 9:**
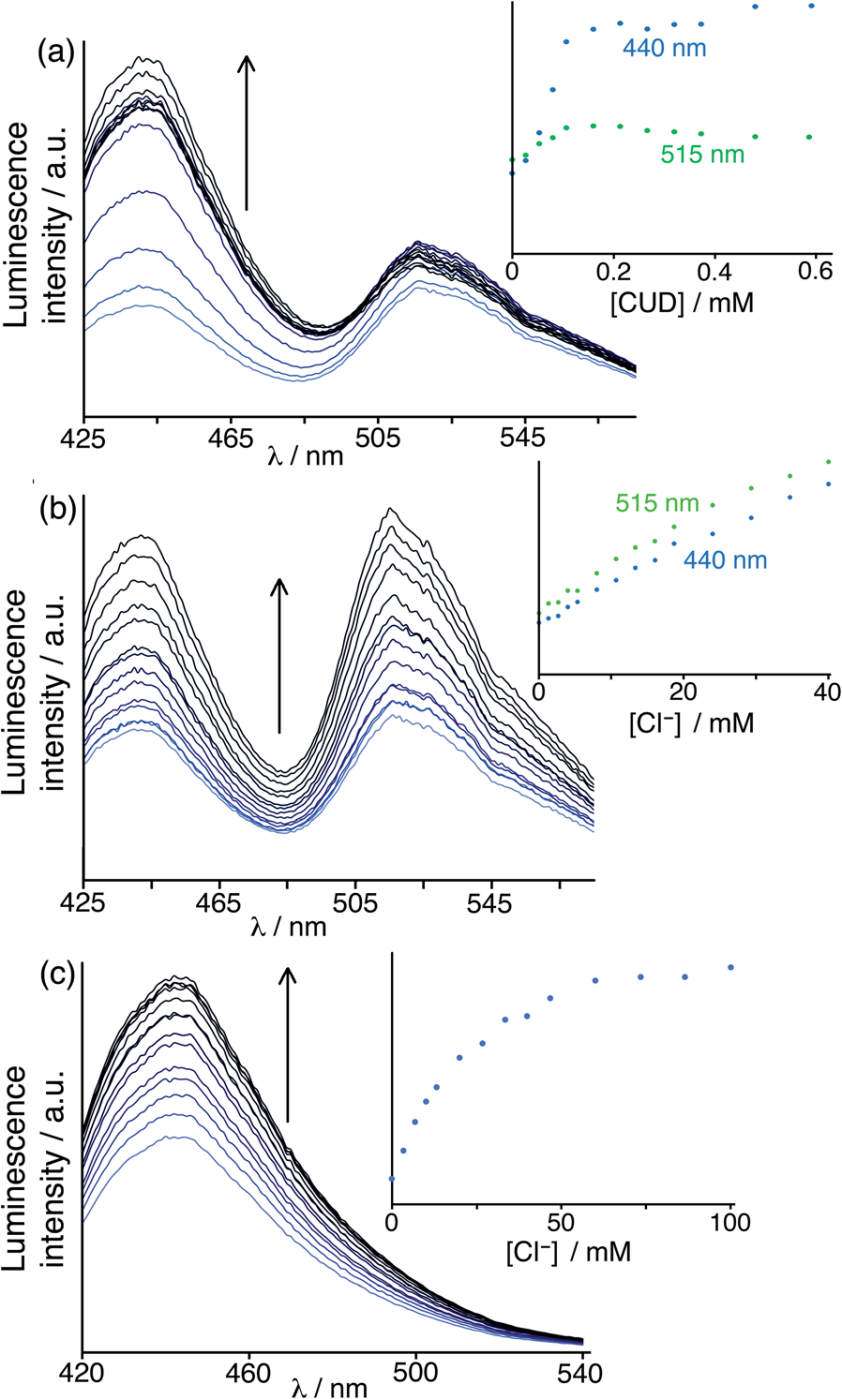
(a) Results of titration of the guest cycloundecanone into a combination of **HW** (150 μM), **FLU** (10 μM) and **MAC** (10 μM) at pH 8 in water (*λ*_exc_ = 395 nm), showing predominantly displacement of cavity-bound **MAC**; (b) results of a similar experiment using chloride as the analyte, unexpectedly showing displacement of *both* cavity-bound **MAC** and surface-bound **FLU**; and (c) a displacement assay experiment using **HW** (150 μM)/**MAC** (10 μM) with added chloride to confirm that chloride can in fact show cavity-binding as well as surface-binding, with the increase in fluorescence from displaced **MAC** fitting to a 1 : 1 binding isotherm (see main text). In each case the intensity changes at the emission maxima during the titration are shown as insets.

The initial assay system was based on the host cage **HW** (150 μM), using the cavity-binding guest **MAC** (10 μM) and the surface-binding fluorophore **FLU** (10 μM), at pH 8 in water. In this case the presence of two fluorophores, which will be affected to different extents by binding of different guest types, will provide the desired ratiometric response: so to start with an additional fixed red-emissive **Ru** component was not used. The un-bound fractions of **MAC** and **FLU** in this mixture gives a combination of blue and green emission components. Fig. 9a shows how addition of portions of the hydrophobic cavity-binding guest cycloundecanone (*K* ≈ 10^6^ M^−1^ for cavity binding in water)^9*a*^ to the above mixture resulted in a rapid increase in the blue component as the **MAC** is progressively displaced from the cage cavity; in contrast the green emission component from surface-bound **FLU** is very little affected. When evaluating the effects of chloride ions, however – expecting chloride to selectively displace **FLU** from the cage surface and boost only the green emission component – we were surprised to observe a clear increase in *both* emission components (Fig. 9b).

**Fig. 10:**
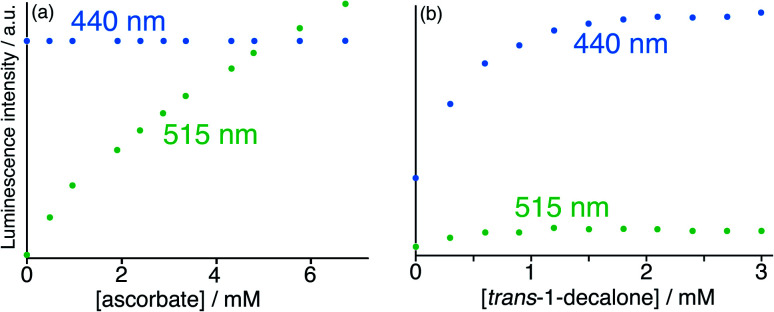
Results of titration of the guests (a) ascorbate and (b) *trans*-1-decalone into a combination of **HW** (150 μM), **FLU** (10 μM) and **MAC** (10 μM) at pH 8 in water (*λ*_exc_ = 395 nm), showing the essentially orthogonal nature of the binding of these two guests and the differing fluorescence responses. Evolution of spectra followed the general appearance shown in Fig. 9; shown here are the peak intensity changes for fluorescence from the **FLU** and **MAC** components during the titrations, from which it is clear that ascorbate selectively displaces **FLU** from the surface of **HW** whereas *trans*-1-decalone selectively displaces **MAC** from the cavity. The colour shift towards green associated with addition of ascorbate and displacement of **FLU** is included in the CIE diagram in Fig. 8c, beginning from ‘*start 2*’.

This implies that, in solution, chloride not only binds to the cage surface and displaces **FLU** (as previously reported),^6^ but is also capable of binding inside the cage cavity and displacing **MAC**. Whilst the majority of the crystal structures we have obtained of salts of **H** or **HW** have the anions occupying only surface portals around the cage and not occupying the central cavity, the crystal structure of triflate salt (reported above, Fig. 6) showed for the first time that cavity-binding of small anions is also possible in the solid state, and therefore it should also be possible in solution. In addition, the fact that binding constants of neutral organic guests tend to be smaller in aqueous solution when chloride is used as the counter-ion^5*b*^ does imply that chloride has the ability to bind both in the cavity and at the cage surface in solution.

To check this we did a FDA analysis using the **HW**/**MAC** pair and adding small portions of chloride in a standard titration (Fig. 9c), and indeed found that chloride could indeed displace **MAC** from the cavity restoring its fluorescence, giving a 1 : 1 cavity-based binding constant of 290 M^−1^ (*cf.* the 1 : 1 binding constant to an individual surface binding site of 750 M^−1^).^6^ This rather unexpected result perfectly confirms the potential value of the dual-mode displacement assay based on two different fluorescence reporters in that it identifies a hitherto unknown mode of binding of chloride in the cage cavity.

In search of an anionic guest that would show surface binding *only* and would have no effect on cavity-bound **MAC**, we considered organic anions which should be too large to bind inside the cavity. Many such anions as their sodium salts (*e.g.* citrate, dodecyl sulfate) immediately resulted in formation of insoluble precipitates with the 16+ **HW** cation. However, titration of portions of sodium ascorbate into the **HW** (150 μM)/**MAC** (10 μM)/**FLU** (10 μM) mixture resulted in a steady increase in the green **FLU** emission component with no significant change in the **MAC** component, indicative of surface-binding only. Fitting this luminescence data to a 1 : 1 binding isotherm, taking account of the presence of two competing surface-binding species, gave a *K* value for 1 : 1 binding at a surface site for ascorbate of 1.3 × 10^4^ M^−1^: significantly weaker than for **FLU** (which is a dianion) but significantly stronger than smaller ions such as halides or nitrate due to the hydrophobic organic backbone.^6^ The fluorescence intensity changes following titration with (surface-binding) ascorbate are shown in Fig. 10a, alongside the essentially orthogonal changes following titration with (cavity-binding) *trans*-1-decalone (Fig. 10b). *This pair of titration results nicely illustrates a key goal of this work: the ability to interrogate binding of different types of guest using two orthogonal recognition processes in a single sensor system*. An illustration on a CIE diagram of the colour change between start and end of the titration with ascorbate (data from Fig. 10a) is included in Fig. 8c, beginning at point ‘start 2’, with an increase in the green emission component as **FLU** is displaced being very clear.

The final optimisation of this dual-mode FDA sensor involved addition of the red-emissive **Ru** component to the cocktail. When we did this initially in the **HW**/**MAC**/**Ru** or **HW**/**FLU**/**Ru** systems, the additional presence of the fixed **Ru**-based red emission allowed the intensity changes from displacement of either **MAC** or **FLU** on their own to be converted to a ratiometric luminescence change with a visible colour shift. In this case however the purpose is different. With both **FLU** and **MAC** present as components of the sensor that respond to orthogonal stimuli there is already a ratiometric response according to analyte type. However, the change in blue/green balance results in small changes of hue against a strongly coloured background: if the same colour change can be shifted towards the centre of the CIE diagram – closer to a white starting point – by addition of a fixed red component, small changes in hue will be more visually obvious.

**Fig. 11:**
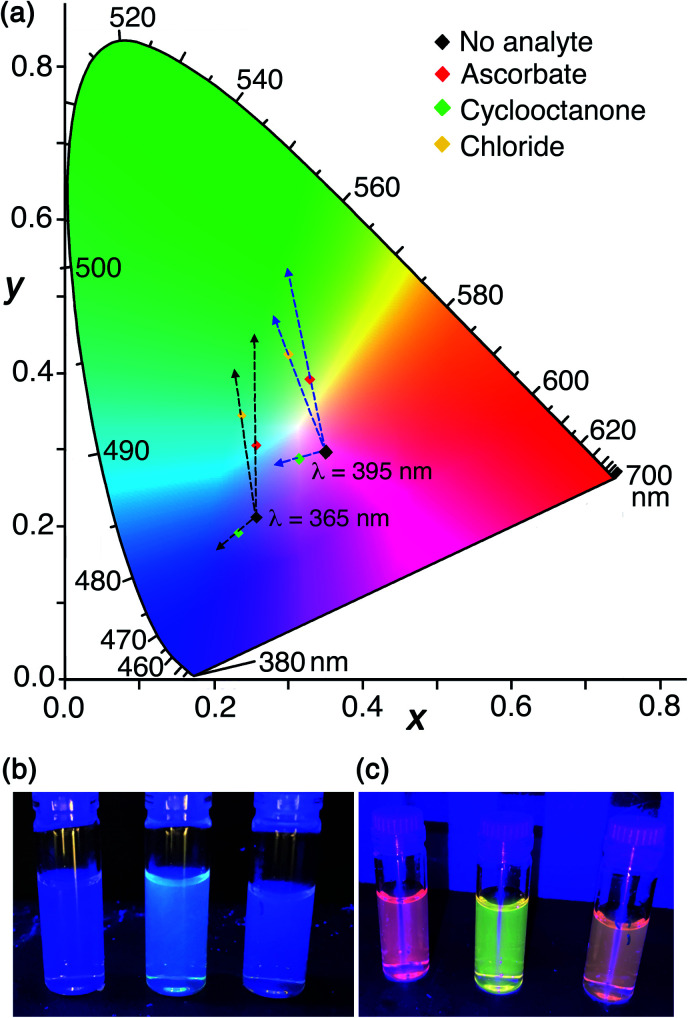
(a) A CIE diagram showing the fluorescence colour changes associated with addition of ascorbate, cyclooctanone, or chloride to a mixture of **HW** (150 μM), **MAC** (10 μM), **FLU** (20 μM) and **Ru** (30 μM). The two sets of dashed arrows starting at different starting points indicate the directions of colour shifts generated by different analytes using 365 nm (black arrows) or 395 nm excitation (blue arrows), respectively. These arrows are just to aid the eye: the end-points of each titration are illustrated by the coloured points. (b and c) Photos of end-points of the titrations with (from left to right) cyclooctanone, chloride and ascorbate taken under (b) 365 nm excitation and (c) 395 nm excitation respectively.

For this final set of experiments, accordingly, we used as the sensor system **HW** (150 μM), **MAC** (10 μM), **FLU** (20 μM) and **Ru** (30 μM): this system again shows nicely the orthogonality of the two independent indicator displacement assays but with the overall colour shifted towards the centre of the CIE colour chart (Fig. 11). The increase in blue emission arising from addition of portions of cyclooctanone (displacing **MAC** from the cavity), and the increase in green emission arising from addition of portions of sodium ascorbate (displacing **FLU** from the surface) are both clear. The resulting colour shifts associated with addition of each analyte are shown on the CIE diagram in Fig. 11a which indicates changes in overall emission colour when using 365 nm excitation. The effect of added chloride, which displaces some of each type of fluorophore given that chloride can participate in both cavity-based and surface-based binding, is a colour shift in an intermediate direction between the responses triggered by ascorbate and cyclooctanone: thus we have a system where we can see immediately the nature of the binding interactions of a guest according to the ‘direction of travel’ of the overall colour change on the CIE diagram. Bromide shows the same mixed response as chloride with a direction of travel of the colour change indicating displacement of mostly surface-bound **FLU** but also a small amount of cavity-bound **MAC**: thus both of these halide anions are capable of some cavity binding which ascorbate clearly is not. Fluoride in contrast showed no cavity binding but displaced only the surface-bound **FLU** fluorophore. We also investigated use of triflate in a similar experiment to see if mixed-mode binding occurs, given the evidence from the crystalline sponge for its ability to bind inside the cavity, but it resulted in precipitation of an insoluble **Hw**/triflate salt.

The visual response can be altered further by using a different excitation wavelength which results in a different balance between the starting R/G/B emission components. The outcome of the same experiment visualised using 395 nm instead of 365 nm excitation is also shown in Fig. 11a using the labelled starting point on the CIE diagram. The difference between using 365 nm and 395 nm excitation in a hand-held lamp to visualise the colour changes is clear: the directions of colour shift on the CIE diagram caused by each added analyte are similar in both cases, but when using 395 nm excitation the difference is more apparent to the eye as the changes start from a point closer to neutral white. This is shown in Fig. 11b and c; in the latter case (395 nm excitation) the visual difference in emission colours associated with addition of cavity-binding or surface-binding guests, or a guest that does both, is more obvious than it is when using 365 nm excitation.

## 3 Conclusions

The ability of the octanuclear cubic host cage to accommodate two different types of guest in different ways (neutral hydrophobic organic guests inside the cavity; anionic guests at the six portals on the cage surface) has been studied both crystallographically (using **H**) and *via* displacement assays in aqueous solution (using **Hw**, bearing water-solubilising substituents). Crystalline sponge experiments have shown that, starting with the ‘empty’ cage **H** (*i.e.* containing solvent molecules in the cavity in the crystalline state) as its fluoroborate salt, it is possible to include both a cavity-binding guest (**MAC**) and any of several different surface-binding anions (iodide, nitrate, sulfate and others) in a single experiment. Taken together with previous work on incorporating cavity-binding guests or surface-binding guests separately into crystals using crystalline sponge experiments, it is clear that the uptake of the two different types of guest into their distinct binding sites in **HW** can be managed independently of one another.

Solution experiments using **HW** with cavity-bound (**MAC**) and surface-bound (**FLU**) fluorescent guests, either individually or in combination, confirmed that these different guest types can be separately displaced by appropriate competing guests, with hydrophobic cavity-binders (*e.g.* cyclic aliphatic ketones) displacing **MAC** and anionic surface-binders (*e.g.* ascorbate, nitrate) displacing **FLU**. This provides the basis of a sensor system which provides two different fluorescence-based responses to two different analyte types in a single scaffold, *i.e.* a ratiometric sensor system that changes the balance between blue and green fluorescence according to where the guest binds. Unexpectedly one anionic guest (chloride) shows the ability to bind in the cage cavity as well as to the surface, and it therefore displaces both fluorophores, providing a fluorescence-based response that is a combination of the responses obtained from ‘pure’ cavity-binders or surface binders. Adding a red emission component in the form of [Ru(bipy)_3_]^2+^ to the system shifts the overall balance of emission colour towards white, with separate R/G/B luminescence components, of which the red component is fixed and the green and blue components are variable. The result is that changes in the blue or green emission components from the ensemble arising from the presence of cavity-binding or surface-binding analytes generates clearly visible and distinct colour changes according to their mode of binding to **HW**; the visibility of the colour changes can also be improved by choice of excitation wavelength with 395 nm excitation showing the difference particularly clearly (Fig. 11c).

We note that there is an interesting conceptual parallel to be drawn between the ‘dual-input, dual-output’ nature of this cage-based analytical system, and molecular logic gates^19^ in which different combinations of chemical stimuli provide the inputs which control the luminescence output (*e.g. p*H and solvent;^20^*p*O_2_ and metal ion concentration;^21^*p*H and *p*O_2_).^22^

## 4 Materials and methods

### 4.1. X-ray crystallography

The crystalline sponge experiments were performed as described in a previous paper,^14^ by immersing pre-grown crystals of **H** (as the tetrafluoroborate salt) into a concentrated MeOH solution containing a mixture of **MAC** and the tetrabutylammonium salt of the relevant anion. Information on the crystal properties, data collections and refinements associated with the structure determinations of the supramolecular complexes of **H** are collected in Table S1† of ESI. The data collections were performed in Experiment Hutch 1 of beamline I-19 at the UK Diamond Light Source synchrotron facility,^23^ using methodology, data processing and software described previously.^14^

### 4.2. Synthesis and spectroscopy

The water-soluble Co_8_ cage **HW** bearing hydroxymethyl substituents that was used for all aqueous solution studies,^9*b*^ and the analogous unsubstituted cage **H** that was used for the crystalline sponge experiments,^15^ were prepared as previously reported. The fluorescent reporters 4-methyl-7-amino-coumarin (**MAC**) and fluorescein (**FLU**) were obtained from Sigma-Aldrich and used as received. Fluorescence measurements were carried out using either a BMG ClarioStar plate reader with 96-well plates, or an Agilent Cary Eclipse fluorimeter. UV/Vis spectra were obtained using an Implen C40 Nanophotometer. The detailed methodology for the fluorescence displacement assay experiments reported in this paper using either surface-binding **FLU**,^6^ or cavity-binding **MAC**,^9*a*^ is described in detail in Section 2 of the ESI.†

## Data availability

Experimental data are in the figures or the ESI.† Raw data in the form of fluorescence spectra during titrations are available in XL format from MDW.

## Author contributions

MDL: synthesis of cages, and all solution spectroscopic measurements of the displacement assays. CGPT: crystalline sponge experiments and X-ray crystallography. MDW: project conception, supervision, and manuscript preparation.

## Conflicts of interest

There are no conflicts to declare.

## Supplementary Material

SC-012-D1SC04272F-s001

SC-012-D1SC04272F-s002

## References

[cit1] Cook T. R., Stang P. J. (2015). Chem. Rev..

[cit2] Fang Y., Powell J. A., Li E., Wang Q., Perry Z., Kirchon A., Yang X., Xiao Z., Zhu C., Zhang L., Huang F., Zhou H.-C. (2019). Chem. Soc. Rev..

[cit3] Sun Y., Chen C., Liu J., Stang P. J. (2020). Chem. Soc. Rev..

[cit4] Grommet A. B., Nitschke J. R. (2017). J. Am. Chem. Soc..

[cit5] Ward M. D., Hunter C. A., Williams N. H. (2018). Acc. Chem. Res..

[cit6] Ludden M. D., Ward M. D. (2021). Dalton Trans..

[cit7] Rizzuto F. J., Wu W. Y., Ronson T. K., Nitschke J. R. (2016). Angew. Chem., Int. Ed. Engl..

[cit8] Sgarlata C., Mugridge J. S., Pluth M. D., Tiedemann B. E. F., Zito V., Arena G., Raymond K. N. (2010). J. Am. Chem. Soc..

[cit9] Turega S., Cullen W., Whitehead M., Hunter C. A., Ward M. D. (2014). J. Am. Chem. Soc..

[cit10] Cullen W., Misuraca M. C., Hunter C. A., Williams N. H., Ward M. D. (2016). Nat. Chem..

[cit11] Sedgwick A. C., Brewster II J. T., Wu T., Feng X., Bull S. D., Qian X., Sessler J. L., James T. D., Anslyn E. V., Sun X. (2021). Chem. Soc. Rev..

[cit12] Hoshino M., Khutia A., Xing H., Inokuma Y., Fujita M. (2016). IUCrJ.

[cit13] Wen K. L., Yu S. S., Huang Z., Chen L. M., Xiao M., Yu X. Q., Pu L. (2015). J. Am. Chem. Soc..

[cit14] Taylor C. G. P., Argent S. P., Ludden M. D., Piper J. R., Mozaceanu C., Barnett S. A., Ward M. D. (2020). Chem.–Eur. J..

[cit15] Tidmarsh I. S., Faust T. B., Adams H., Harding L. P., Russo L., Clegg W., Ward M. D. (2008). J. Am. Chem. Soc..

[cit16] Metherell A. J., Ward M. D. (2016). Dalton Trans..

[cit17] Pike S. J., Hutchinson J. J., Hunter C. A. (2017). J. Am. Chem. Soc..

[cit18] Metherell A. J., Curty C., Zaugg A., Saad S. T., Dennison G. H., Ward M. D. (2016). J. Mater. Chem. C.

[cit19] Magri D. C. (2021). Coord. Chem. Rev..

[cit20] Trifoi L. A., Hodgson G. K., Dogantzis N. P., Impellizzeri S. (2020). Front. Chem..

[cit21] de Sousa M., Kluciar M., Abad S., Miranda M. A., de Castro B., Pischel U. (2004). Photochem. Photobiol. Sci..

[cit22] Gunnlaugsson T., MacDónail D. A., Parker D. (2000). Chem. Commun..

[cit23] Allan D. R., Nowell H., Barnett S. A., Warren M. R., Wilcox A., Christensen J., Saunders L. K., Peach A., Hooper M. T., Zaja L., Patel S., Cahill L., Marshall R., Trimnell S., Foster A. J., Bates T., Lay S., Williams M. A., Hathaway P. V., Winter G., Gerstel M., Wooley R. W. (2017). Crystals.

