# Supplementary material for: Orthogonal binding and displacement of different guest types using a coordination cage host with cavity-based and surface-based binding sites
Source: Chem Sci. 2021 Aug 25;12(38):12640–50. doi: 10.1039/d1sc04272f (PMC8494021; doi:10.1039/d1sc04272f)
Supplement: SC-012-D1SC04272F-s001 [file SC-012-D1SC04272F-s001.pdf]

Electronic Supplementary Material (ESI) for Chemical Science.
This journal is © The Royal Society of Chemistry 2021

Orthogonal binding and displacement of different guest types using a coordination cage host with cavity-based and surface-based binding sites

M. D. Ludden, C. G. P. Taylor and M. D. Ward

Supporting information

1. Crystallographic details

Details of the crystals used, data collection and refinements are given in Table S1 (overleaf). As is usual with crystallographic structure determinations of this kind on elaborate supramolecular assemblies, scattering is weak and refinement problems are significant due to substantial disorder, principally of anions and solvent molecules, but also – in these cases – of cavity-bound guests. The cage superstructure itself is generally well-behaved in all cases. These problems required (i) extensive use of restraints to ensure geometrically reasonable structures, and (ii) elimination of regions of diffuse electron density using the solvent mask feature in OLEX leaving apparent voids in the lattices. Details pertaining to each structure are included in the individual CIFs. Discussion of the structures is accordingly at the level of demonstrating the presence of particular anion types around the cage surface, and the presence (or not) of an organic guest in the cavity with an approximate site occupancy; detailed discussion of structural minutiae is kept to a minimum.

CCDC deposition numbers: 2101190-2101194

Table S1. Summary of crystallographic data for the five crystal structures.[a]

Structure name	**H•MAC•I**	**H•MAC•NO_3**	**H•MAC•PF_6**	**H•MAC•SO_4**	**H•CF_3SO_3**
CCDC number	2101191	2101192	2101190	2101193	2101194
Empirical formula	$C_{383}Co_8H_{421}I_{16}N_{73}O_{39}$	$B_3C_{375}Co_8F_{12}H_{379.9}N_{86.1}O_{69.2}$	$C_{389}Co_8F_{96}H_{414}N_{74}O_{37}P_{16}$	$BC_{379}Co_8F_4H_{405}N_{73}O_{65}S_{7.5}$	$C_{372.49}Co_8F_{48}H_{342.99}N_{72}O_{68.49}S_{16}$
Formula weight	7713.67	7932.00	9508.88	7821.44	8820.28
Crystal system	monoclinic	monoclinic	monoclinic	monoclinic	trigonal
Space group	*C*2/c	*C*2/c	*C*2/c	*C*2/c	*R*-3
a/Å	32.9277(2)	32.7342(4)	34.60663(19)	33.1430(5)	61.6062(5)
b/Å	30.0730(2)	29.9459(4)	29.36102(18)	29.6976(5)	61.6062(5)
c/Å	40.7670(3)	40.2590(7)	41.9366(4)	39.3556(8)	38.3057(4)
β/°	95.5760(10)	95.7000(10)	97.2150(7)	96.4465(17)	90.0
Volume/$Å^3$	40177.9(5)	39269.0(10)	42273.8(4)	38491.6(11)	125905.0(16)
Z	4	4	4	4	12
ρ_{calc} / g cm^3	1.275	1.342	1.494	1.350	1.396
μ/mm^{-1}	1.317	0.389	0.446	0.428	0.451
Crystal size/mm^3	0.08 × 0.05 × 0.04	0.12 × 0.11 × 0.08	0.1 × 0.09 × 0.06	0.1 × 0.08 × 0.07	0.1 × 0.08 × 0.07
2Θ range for data collection/°	3.24 to 59.894	3.292 to 59.894	1.898 to 58.044	3.338 to 59.894	1.268 to 51.006
Reflections collected	352450	343424	321209	338727	517504
Independent reflections / R_{int}	64020 / 0.0634	62520 / 0.0555	61669 / 0.0557	61248 / 0.0639	57192 / 0.0842
Data/restraints/parameters	64020/7039/2152	62520/7876/2410	61669/12076/2910	61248/7233/2175	57192/5148/3145
Goodness-of-fit on F^2	1.079	1.166	1.275	1.233	1.306
Final R_1 / wR_2 [b]	0.0644 / 0.2289	0.0943 / 0.3364	0.1033 / 0.3670	0.1076 / 0.3732	0.1208 / 0.4006
Largest diff. peak/hole / e $Å^{-3}$	2.67/-1.63	2.01/-0.59	2.88/-1.59	1.59/-1.01	1.41/-0.81

a Common to all structure determinations: synchrotron radiation (λ = 0.6889 Å); *T* = 100K
b R_1 based on [$I \geq 2\sigma(I)$]; wR_2 based on all data

2. Experimental methodology for fluorescence-based displacement assays

*a) Displacement of **MAC** in a **H^W**/**MAC**/[Ru(bpy)$_3$]$^{2+}$ system*

Displacement titrations to determine the effects of different analytes were performed starting with two solutions: one containing **MAC**, [Ru(bpy)$_3$]$^{2+}$ and **H^W**; and the other containing **MAC**, [Ru(bpy)$_3$]$^{2+}$, **H^W** and the chosen analyte. These were prepared by first making a solution of **MAC**, [Ru(bpy)$_3$]$^{2+}$ and **H^W** at double the desired concentration (which are in the main text), which was then split into two vials. One was diluted 1:1 with deionised water, whilst the other was diluted with a premade analyte solution such that the concentrations of **MAC**, [Ru(bpy)$_3$]$^{2+}$ and **H^W** were consistent in both solutions (typically 10 µM, 30 µM and 150 µM, respectively) but one contained analyte and the other did not. These solutions were then mixed in varying ratios to a total volume of 200 µL such that the concentrations of **MAC**, [Ru(bpy)$_3$]$^{2+}$ and **H^W** were fixed but the concentration of analyte varied from one well to the next. These titrations used between 12 and 18 wells of a standard 96-well plate per experiment, and a minimum of two repeats was carried out for each titration. The plate-reader instrument was equilibrated to 298 K and an excitation wavelength of 395 nm was used for these experiments.

*b) Displacement of **FLU** in a **H^W**/**FLU**/[Ru(bpy)$_3$]$^{2+}$ system*

These experiments were set up using the same method as described in part a) but with the initial solution containing **FLU**, [Ru(bpy)$_3$]$^{2+}$ and **H^W** at 10 µM, 100 µM and 160 µM, respectively, using 50 mM borate buffer at pH 8.5 as solvent given the requirement for **FLU** to be doubly deprotonated. The rest of the procedure (splitting this stock solution in half; diluting one by 2x using only buffer solution; dilutin the other by 2x using buffer solution also containing the relevant analyte; and then combing these two solutions in different proportions such that the analyte concentration varied) was followed as described above. The plate-reader instrument was equilibrated to 298 K and an excitation wavelength of 450 nm was used for these experiments.

*c) Displacement of **MAC** or **FLU** in a **H^W**/**MAC**/**FLU** system*

The basic methodology again involved a solution containing **MAC**, **FLU** and **H^W** at double the required concentration for titrations (*i.e.* initial concentrations of 20 µM for **MAC** and **FLU**, 300 µM for **H^W**). This was split into two solutions, each of which was diluted by a factor two as described above, such that one solution contained the analyte and the other did not. Owing to the presence of **FLU** in these solutions the solutions were made at pH 8.5 using 50 mM borate buffer. Combination of these two solutions in different proportions to vary the analyte concentration was effected as described above. The plate-reader instrument was equilibrated to 298 K and an excitation wavelength of 395 nm was used for these experiments.

*d) Displacement of **MAC** or **FLU** in a **H^W**/**MAC**/**FLU**/[Ru(bpy)$_3$]$^{2+}$ system (RGB emissive components)*

Experiments in which [Ru(bpy)$_3$]$^{2+}$, FLU and MAC were all present as emissive components were carried out through preparation of a solution containing all four components (**MAC**, **FLU**, [Ru(bpy)$_3$]$^{2+}$ and **H^W**) at double the desired concentration (*i.e.* initial concentrations of 20 µM **MAC**, 40 µM **FLU**, 60 µM [Ru(bpy)$_3$]$^{2+}$ and 300 µM **H^W** before dilution). This sample included 50 mM borate buffer at pH 8.5. This was then split into two

samples and diluted 1:1 using deionised water for one sample, and a solution of the analyte under investigation for the other sample. As before, these solutions were then mixed in varying ratios to a volume of 200 µL so as to increase the concentration of analyte from one well to the next. These titrations used between 11 and 14 wells of a standard 96-well plate per experiment, and a minimum of two repeats was carried out for each titration. Due to the number of individual components within this system, the instrument was heated to 308 K for 20 minutes before equilibrating at 298 K to carry out the measurements, to ensure thorough mixing. An excitation wavelength of 395 nm was used for these experiments.

e) Conversion of luminescence spectra to CIE coordinates

After recording a fluorescence spectrum for each well of any given titration, the spectra obtained from wells containing no analyte and the highest concentration of analyte (termed 'start' and 'end' within the results and discussion section of this article) were converted to sets of (*x,y*) chromaticity coordinates in the CIE-1931 colour space. This was done using the methodology here:

(https://en.wikipedia.org/wiki/CIE_1931_color_space#CIE_xy_chromaticity_diagram_and_the_CIE_xyY_color_space; last accessed August 2021)

by multiplying the emission spectra intensity at each wavelength with the colour-matching function for the CIE-1931 colour space (obtained from http://www.cvrl.org/ – last accessed August 2021) in an Excel spreadsheet.
